# Diagnostic performance of the Brief Cognitive Screening Battery-Indonesian version in detecting cognitive impairment

**DOI:** 10.1055/s-0045-1809335

**Published:** 2025-06-01

**Authors:** Fasihah Irfani Fitri, Dina Nazriani, Octaviasari Agatha Dachi, Ricardo Nitrini, Paulo Caramelli

**Affiliations:** 1 Universitas Sumatera Utara, Faculty of Medicine, Department of Neurology, Medan, Indonesia.; 2 Universitas Sumatera Utara, Faculty of Psychology, Medan, Indonesia.; 3 Universitas Sumatera Utara, Faculty of Medicine, Medan, Indonesia.; 4 Universidade de São Paulo, Faculdade de Medicina, Departamento de Neurologia, São Paulo SP, Brazil.; 5 Universidade Federal de Minas Gerais, Faculdade de Medicina, Grupo de Pesquisa em Neurologia Cognitiva e do Comportamento, Belo Horizonte MG, Brazil.

**Keywords:** Brief Cognitive Screening Battery, Cognition, Dementia, Indonesia

## Abstract

**Background:**

Neuropsychological and functional assessments are crucial for identifying the transition from healthy aging to dementia. While brief cognitive batteries have become popular for their practicality, most have been developed in high-income countries, neglecting the diverse educational backgrounds found in developing nations.

**Objective:**

This study focuses on the Brief Cognitive Screening Battery (BCSB) adapted for Indonesia (BCSB-INA), aiming to investigate its diagnostic accuracy in detecting cognitive impairment among older adults.

**Methods:**

This cross-sectional study was conducted at the Memory Clinic of Universitas Sumatera Utara Hospital from January to August 2024, including participants aged 50 and above. Subjects underwent cognitive assessments using MoCA-INA and BCSB-INA. Data analysis involved ROC curves to evaluate the tests' accuracy.

**Results:**

A total of 140 subjects were included, with significant differences in cognitive test scores between those with cognitive impairment and normal individuals. The BCSB-INA demonstrated good diagnostic performance, with an AUC of 0.875 when including the Clock Drawing Test (CDT) and 0.810 without it. The development of a multivariate model further enhances its diagnostic capabilities, allowing for more tailored intervention strategies.

**Conclusion:**

The BCSB-INA represents an important improvement in cognitive assessment for older adults in Indonesia, showing good sensitivity and specificity. Continued research and updates to cognitive assessment tools are crucial to meet the increasing demand for effective dementia screening in diverse populations.

## INTRODUCTION

Neuropsychological and functional assessments are essential for identifying the transition from healthy aging to dementia. These assessments provide valuable insights into cognitive decline and help in the early detection of dementia, allowing for timely interventions and management strategies. 1 Among the various assessment tools available, brief cognitive batteries have gained popularity for their practicality in clinical and research settings. 2 Despite their widespread use, most cognitive assessment instruments have been developed and validated in high-income countries, where the populations often have more uniform educational backgrounds and cognitive profiles. 3 This limitation is particularly pronounced in low- or middle-income countries, where socioeconomic factors contribute to disparities in education, literacy, and health literacy. 4 Such disparities underscore the need for cognitive assessment tools that are culturally and educationally appropriate for diverse populations. Moreover, as dementia screening becomes increasingly vital in both epidemiological studies and routine clinical practice, the emphasis on brief cognitive batteries that can be administered efficiently is paramount.

The existing batteries often overlook the unique challenges faced by populations with low education and literacy, which are common in developing countries. 5 Therefore, a growing body of research is focusing on creating cognitive assessment tools that are not only robust but also adaptable to varying educational levels. Previous studies have shown promising results, indicating that cognitive batteries specifically designed for illiterate or low-educated populations can perform well in detecting cognitive impairment. The Brief Cognitive Screening Battery (BCSB), developed by Nitrini et al. 6 has been used in populations with diverse educational backgrounds, including illiterate people, and has been proven to be highly accurate in diagnosing dementia in community and clinical settings. 7 8 9 10 11 It has been culturally adapted in Bahasa Indonesia, called BCSB-INA, to denote the Indonesian version, and was employed in community-dwelling older adults in North Sumatra, Indonesia, to assess its feasibility and obtain normative data. This adaptation has proven to be well understood and feasible for use among older adults with diverse educational backgrounds in North Sumatra. 12 Therefore, this study aimed to investigate the diagnostic accuracy of the BCSB-INA in detecting cognitive impairment in older adult individuals.

## METHODS

### Study design and subjects

This was a cross-sectional study conducted at the Memory Clinic Universitas Sumatera Utara Hospital in Medan from January to August 2024. We included subjects aged 50 years or above who were able to communicate in Bahasa Indonesia and had an informant present at the time of assessment to give objective information about daily life activities and functional status. We excluded subjects who had severe uncorrected visual and hearing impairments that could interfere with the assessments. The research was carried out following ethical guidelines and received approval from the Ethics Committee of the Faculty of Medicine at Universitas Sumatera Utara (No. 540/KEPK/USU/2024). All subjects provided informed consent before taking part in the study.

### Procedures

All subjects underwent general, neurology, and cognitive assessments using MoCA-INA and BCSB-INA. The MoCA assesses various cognitive domains, including visuospatial/executive function, naming, memory, attention, language, abstraction, delayed recall, and orientation to time and place. Scores range from 0 to 30, with higher scores indicating better cognitive performance, and an extra point is given to those with 12 or fewer years of education. 13 The MoCA has been transcultural validity to Bahasa Indonesia, then called MoCA-INA, and has been used widely in Indonesia, both in community, as well as in clinical settings. 14 We determined the presence of cognitive impairment using age- and education-adjusted cutoffs of MoCA-INA specific to the Indonesian context. 15

The BCSB-INA is the Indonesian version of BCSB. The details of cultural adaptation and validity process can be found elsewhere. 12 The BCSB-INA was administered to all subjects and controls using a sheet displaying 10 pictures. Subjects were asked to name each drawing, which was the naming part. After naming, the sheet was removed, and subjects recalled the drawings (incidental memory). The sheet was then shown again for 30 seconds for memorization, followed by recall to score immediate memory and a learning test. After an interference period involving a category fluency test and a clock drawing test (CDT), subjects recalled as many items as possible in one minute for the delayed recall test. Lastly, a recognition test was conducted with a sheet of 20 drawings, including 10 previously shown objects and 10 distractors; scores were calculated by subtracting incorrect responses from correct ones. 10

### Data analysis

Mean comparisons of demographic data were conducted using either the t-test or chi-square test as appropriate. For comparing test scores between subjects with and without cognitive impairment, the t-test or Mann-Whitney test was employed. A significance level of 0.05 was accepted. Statistical analyses were performed using the SPSS software package 22. ROC (receiver operator characteristic) analysis was used to evaluate the accuracy of each test.

## RESULTS

A total of 140 subjects were included in this study. Demographic data of subjects with cognitive impairment and normal individuals are shown in Table 1 .

**Table 1:** Subject characteristics

Characteristic		Cognitively Impaired ( *n* = 74)	Normal ( *n* = 66)	*p*
Age (years), mean (SD)		67.69 (6.83)	66.68 (8.42)	0.716 ^a^
Sex	Male	31	43	0.006 ^b^
	Female	43	23	
Educational status	Did not attend school	−	1	0.001 ^b^
	Elementary school	14	3	
	Junior high school	16	3	
	Senior high school	14	21	
	University	30	38	
Occupational status	Unemployed/housewife/retired	55	44	0.343 ^b^
	Employed	4	8	
	Self-employed	15	14	

Abbreviation: SD, standard deviation.

Notes: ^a^ Independent sample t-test; ^b^ Chi-Square.

Significant differences were observed in test scores between subjects with cognitive impairment and normal individuals on both the MoCa-INA (p < 0.05) and BCSB-INA (p < 0.05) assessments, as shown in Table 2 .

**Table 2:** Scores of cognitive tests

Variables	Cognitively Impaired ( *n* = 74) Mean (SD)	Normal ( *n* = 66) Mean (SD)	*p*
**MoCA-INA**	**11.62 (6.232)**	**23.15 (2.227)**	**<0.001**
Visuospatial/executive	2.04 (1.565)	3.98 (1.030)	<0.001
Naming	1.96 (1.091)	2.79 (0.512)	<0.001
Attention	2.78 (1.932)	5.08 (1.042)	<0.001
Language	0.97 (0.965)	2.45 (0.661)	<0.001
Abstraction	0.47 (0.579)	1.52 (0.588)	<0.001
Delayed recall	0.15 (0.395)	1.55 (1.438)	<0.001
Orientation	3.24 (2.22)	5.79 (0.541)	<0.001
**BCSB INA**			
Naming	8.55 (2.98)	10 (0)	<0.001
Incidental memory	3.14 (2.366)	5.42 (1.468)	<0.001
Learning	4.76 (3.033)	7.79 (1.409)	<0.001
Delayed recall	5.39 (3.23)	8.59 (1.588)	<0.001
Recognition	4.12 (3.664)	7.91 (1.895)	<0.001
Verbal fluency	8.15 (3.403)	9.82 (0.959)	<0.001
Clock drawing	13.14 (8.413)	26.17 (8.947)	<0.001

**Figure 1:**
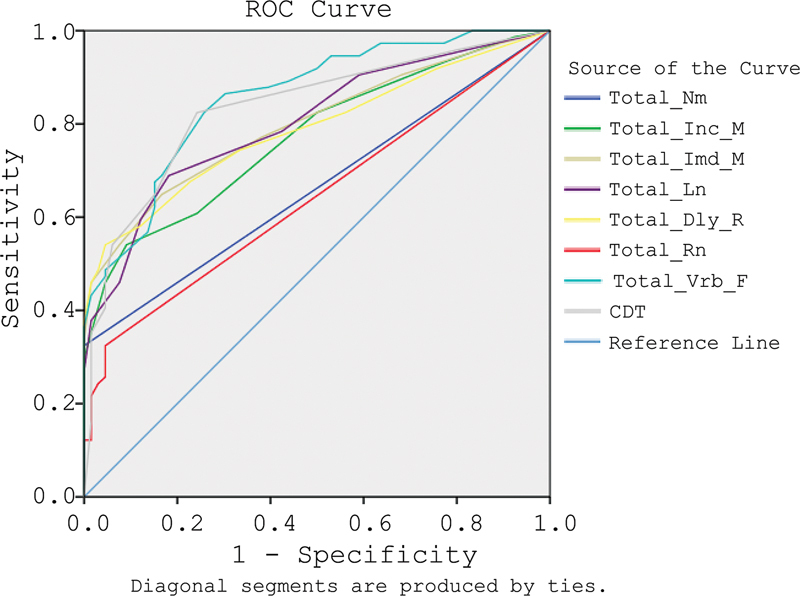
The ROC Curve.

Table 3 presents the Area Under the Curve (AUC), cut-off values, sensitivity, and specificity for the BCSB-INA scores that were generated using ROC analysis ( Figure 1 ). The combined results of the seven dimensions of the BCSB-INA, along with the Clock Drawing Test (CDT), demonstrate good discriminatory capabilities, as indicated by the AUC values. Specifically, the dimensions are ranked from most to least discriminative as follows:

**Table 3:** Cut-off scores of the tests for the whole subjects

	Area under the curve	Lower Bound 95% CI	Upper Bound 95% CI	Cut-off scores	Sensitivity (%)	Specificity (%)
Naming	0.662	0.572	0.752	9.5	32.4	100
Incidental memory	0.772	0.695	0.848	3.5	54.1	90.9
Immediate memory	0.799	0.726	0.872	6.5	64.9	83.3
Learning	0.802	0.730	0.874	7.5	68.9	81.8
Delayed recall	0.788	0.713	0.863	4.5	54.1	95.5
Recognition	0.641	0.550	0.732	9.5	32.4	95.5
Verbal fluency	0.857	0.797	0.917	20.5	82.4	74.2
Clock drawing	0.834	0.766	0.902	8.5	82.4	75.8

- verbal fluency,
- CDT,
- learning,
- immediate memory,
- delayed recall,
- incidental memory,
- naming, and
- recognition.

Notably, verbal fluency emerged as the most effective measure for distinguishing between disorder and normal subjects. Additionally, the BCSB-INA test shows a high AUC value of 0.875 when including the CDT and 0.810 without it, indicating its good capability to differentiate between the two groups.

Multivariate analysis selected CDT, verbal fluency, delayed recall, learning, and immediate memory as having sufficiently good AUC, sensitivity, and specificity to produce the new model below:





A lower score indicates a higher likelihood of cognitive impairment, while a higher score suggests a lower likelihood. In retesting the same sample, the new model achieved an AUC of 0.878, demonstrating improved capacity to distinguish between the cognitively impaired and normal individuals.

## DISCUSSION

The results of this study highlight the diagnostic performance of the BCSB-INA as a valuable tool for detecting cognitive impairment among older adults. Our findings indicate that the BCSB-INA demonstrates good sensitivity and specificity, crucial parameters in evaluating the effectiveness of any diagnostic test. The use of ROC analysis allowed us to assess the BCSB-INA's ability to distinguish between cognitively impaired and normal individuals. The AUC values derived from this analysis are particularly noteworthy. With an AUC of 0.875 when including the Clock Drawing Test (CDT) and 0.810 without it, the BCSB-INA showcases a high level of diagnostic accuracy. An AUC value above 0.8 is generally considered excellent, indicating that the BCSB-INA is highly effective in distinguishing between the two groups. This robust performance is further supported by the specific sensitivity and specificity values of various cognitive domains assessed by the BCSB-INA. The high specificity values across several dimensions – such as delayed recall (95.5%) and incidental memory (90.9%) – suggest that these tests are effective in accurately identifying individuals without cognitive impairment. High specificity is crucial in clinical settings, as it minimizes the likelihood of false positives, allowing healthcare providers to focus resources on those who genuinely require further assessment or intervention.

Our study identified a cutoff of ≤ 4.5 for the delayed recall subtest, which aligns closely with the commonly used cutoff of ≤ 5. 10 This finding is consistent with earlier research utilizing the BCSB across various clinical settings and among individuals with different educational backgrounds to diagnose dementia. A matched subset from a previous study among 100 dementia patients showed that the delayed recall of BCSB effectively distinguished mild to moderate dementia from controls, with a sensitivity of 93.3% and specificity of 96.6% at a cut-off score of ≤5, leading to its increased use as a diagnostic tool for dementia and memory impairment in clinical settings. 7 In a study of 73 patients with mild Alzheimer's dementia and 94 higher-educated controls, the BCSB's effectiveness for diagnosing Alzheimer's was assessed. The delayed recall of the BCSB had the highest area under the ROC curve (AUROC) at 0.931, with a sensitivity of 82.2% and specificity of 90.4% at a cut-off of ≤5. Additionally, a mathematical model using three subtests – delayed recall and learning, plus verbal fluency (animals in one minute) – achieved a strong accuracy with an AUROC of 0.917. 9 Fichman-Charchat et al. examined the BCSB for diagnosing mild Alzheimer's dementia in a geriatric outpatient unit, comparing Alzheimer's patients with a non-Alzheimer's group. The delayed recall proved to be the most effective diagnostic tool, with sensitivity and specificity above 80%, although the cut-off score was lower (<5) due to several factors. 8 In 2006, Takada et al. compared the effectiveness of two memory tests – the CERAD Word List delayed recall and the delayed recall of the BCSB – for diagnosing dementia. The delayed recall from BCSB demonstrated a trend of higher accuracy across the entire sample, suggesting it could be a valuable alternative for diagnosing dementia in illiterate populations. 16 A recent study assessed the BCSB effectiveness in distinguishing Alzheimer's disease (AD), non-AD cognitive impairment, and healthy cognition among 117 participants. This included 45 with MCI or mild dementia within the AD continuum (positive amyloid biomarker or A + ), 27 with non-AD diagnoses (A-), and 45 cognitively healthy controls. The delayed recall of the FMT (DR-FMT) achieved high accuracy for differentiating AD and non-AD from controls (AUROC = 0.87) and for distinguishing AD from controls (AUROC = 0.91). 17

In our study, a significant distinction was observed between individuals with cognitive impairment and normal subjects in terms of educational status, as shown in Table 1 . Specifically, more individuals with cognitive impairment have attended elementary school or junior high school compared to those in the normal group. On the other hand, the normal group has a higher representation of individuals who have attended senior high school or university. The significant difference in educational status suggests that education may play an important role in cognitive health, as reported in Indonesia 18 19 and globally, 20 supporting the cognitive reserve hypothesis. 21 Previous studies have consistently shown that individuals with higher levels of education tend to have a better capacity to cope with cognitive decline, potentially due to more robust neural networks that are built over years of learning. 21 22

### Clinical implications

The good diagnostic performance of the BCSB-INA indicates that it can serve as a practical and reliable screening tool in various settings, particularly in areas where traditional assessments may not be feasible due to educational or cultural barriers. The emphasis on cognitive domains, such as delayed recall, which showed the highest sensitivity and specificity, suggests that these could be prioritized in screening protocols. Furthermore, the development of a multivariate model that incorporates key cognitive dimensions into a single score enhances diagnostic capabilities. By utilizing this model, clinicians can gain a nuanced understanding of an individual's cognitive status, enabling more tailored and effective intervention strategies. The new model's AUC of 0.878 upon retesting indicates a good potential for improved diagnostic performance, further validating the BCSB-INA as a tool for ongoing cognitive assessment.

### Limitations and future directions

While the BCSB-INA's diagnostic performance is promising, it is essential to acknowledge the study's limitations. The sample size, though adequate for initial validation, may not capture the full diversity of cognitive profiles present in the broader Indonesian population. Future studies should aim to include larger and more heterogeneous samples to enhance the generalizability of the findings. Longitudinal studies would also be beneficial to assess the BCSB-INA's effectiveness in tracking cognitive decline over time.

In conclusion, the BCSB-INA represents a significant advancement in cognitive assessment for older adults in Indonesia. Its good sensitivity, specificity, and overall diagnostic performance, as evidenced by ROC analysis, underscore its potential as a vital tool in the early detection of cognitive impairment and dementia. Continued research and adaptation of such tools will be crucial in addressing the growing need for effective dementia screening in diverse populations.

## References

[1] Velayudhan L Ryu S H Raczek M Review of brief cognitive tests for patients with suspected dementia Int Psychogeriatr 2014 26 08 1247 1262 10.1017/S1041610214000416 24685119 PMC4071993

[2] Mattke S Batie D Chodosh J Expanding the use of brief cognitive assessments to detect suspected early-stage cognitive impairment in primary care Alzheimers Dement 2023 19 09 4252 4259 10.1002/alz.13051 37073874

[3] Chun C T Seward K Patterson A Melton A MacDonald-Wicks L Evaluation of Available Cognitive Tools Used to Measure Mild Cognitive Decline: A Scoping Review Nutrients 2021 13 11 3974 10.3390/nu13113974 34836228 PMC8623828

[4] Garuma D Lamba D Abessa T G Bonnechère B Advancing public health: enabling culture-fair and education-independent automated cognitive assessment in low- and middle-income countries Front Public Health 2024 12 1.377482E6 10.3389/fpubh.2024.1377482 PMC11239414 39005983

[5] Bernstein Sideman A Al-Rousan T Tsoy E Facilitators and Barriers to Dementia Assessment and Diagnosis: Perspectives From Dementia Experts Within a Global Health Context Front Neurol 2022 13 769360 10.3389/fneur.2022.769360 35418934 PMC8997042

[6] Nitrini R Lefèvre B H Mathias S C [Neuropsychological tests of simple application for diagnosing dementia] Arq Neuro-Psiquiatr 1994 52 04 457 465 10.1590/S0004-282Doi: × 1994000400001 7611936

[7] Nitrini R Mathias S C Caramelli P Evaluation of 100 patients with dementia in São Paulo, Brazil: correlation with socioeconomic status and education Alzheimer Dis Assoc Disord 1995 9 03 146 151 8534413

[8] Fichman-Charchat H Miranda C V Fernandes C S Brief Cognitive Screening Battery (BCSB) is a very useful tool for diagnosis of probable mild Alzheimeŕs disease in a geriatric clinic Arq Neuro-Psiquiatr 2016 74 02 149 154 10.1590/0004-282Doi: × 20150202 26690839

[9] Nitrini R Caramelli P Porto C S Brief cognitive battery in the diagnosis of mild Alzheimer's disease in subjects with medium and high levels of education Dement Neuropsychol 2007 1 01 32 36 10.1590/S1980-57642008DN10100006 29213365 PMC5619381

[10] Nitrini R Bucki S MD Yassuda M S Fichman H C Caramelli P The Figure Memory Test: diagnosis of memory impairment in populations with heterogeneous educational background Dement Neuropsychol 2021 15 02 173 185 10.1590/1980-57642021dn15-020004 34345358 PMC8283874

[11] Yassuda M S da Silva H S Lima-Silva T B Normative data for the Brief Cognitive Screening Battery stratified by age and education Dement Neuropsychol 2017 11 01 48 53 10.1590/1980-57642016dn11-010008 29213493 PMC5619214

[12] Fitri F I Naci L Turana Y Modified Brief Cognitive Screening Battery - Indonesian Version: cross-cultural adaptation and normative data based on demographic factors in North Sumatra, Indonesia Front Neurol 2024 14 1.306356E6 10.3389/fneur.2023.1306356 PMC10822921 38288332

[13] Nasreddine Z S Phillips N A Bédirian V The Montreal Cognitive Assessment, MoCA: a brief screening tool for mild cognitive impairment J Am Geriatr Soc 2005 53 04 695 699 10.1111/j.1532-5415.2005.53221.x 15817019

[14] Husein N Silvia L Yetty R. dkk.Uji Validitas dan Reabilitas Montreal Cognitive Assessment Versi Indonesia (MoCA-Ina) Untuk Skrining Gangguan Fungsi Kognitif Neurona Neuro Sains 2010 27 04 15 22

[15] Prasetyo B T Lumempouw S F Ramli Y Herqutanto. Nilai normal Montreal Cognitive Assessment versi Indonesia Neurona 2011 29

[16] Takada L T Caramelli P Fichman H C Comparison between two tests of delayed recall for the diagnosis of dementia Arq Neuro-Psiquiatr 2006 64 01 35 40 10.1590/s0004-282Doi: × 2006000100008 16622550

[17] Peles P RH Salvador L S Souza L C Caramelli P Accuracy of the Brief Cognitive Screening Battery for diagnosing Alzheimer's disease defined by cerebrospinal fluid biomarkers and AT(N) classification: a case-control study Arq Neuro-Psiquiatr 2022 80 01 23 29 10.1590/0004-282X-ANP-2021-0012 PMC9651505 34816970

[18] Ong P A Annisafitrie F R Purnamasari N Dementia Prevalence, Comorbidities, and Lifestyle Among Jatinangor Elders Front Neurol 2021 12 643480 10.3389/fneur.2021.643480 34367043 PMC8345013

[19] Fitri F I Farina N Turana Y Modifiable risk factors for dementia in Indonesia: Results from STRiDE project Neurol Asia 2023 28 04 1009 1017

[20] Livingston G Huntley J Liu K Y Dementia prevention, intervention, and care: 2024 report of the Lancet standing Commission Lancet 2024 404(10452):572 628 10.1016/S0140-6736(24)01296-0 39096926

[21] Stern Y Cognitive reserve in ageing and Alzheimer's disease Lancet Neurol 2012 11 11 1006 1012 10.1016/S1474-4422(12)70191-6 23079557 PMC3507991

[22] Meng X D'Arcy C Education and dementia in the context of the cognitive reserve hypothesis: a systematic review with meta-analyses and qualitative analyses PLoS One 2012 7 06 e38268 10.1371/journal.pone.0038268 22675535 PMC3366926

